# Prefrontal FGF1 Signaling is Required for Accumbal Deep Brain Stimulation Treatment of Addiction

**DOI:** 10.1002/advs.202413370

**Published:** 2025-02-27

**Authors:** Wan‐Kun Gong, Xue Li, Le Wang, Qian Yang, Alix Tiran‐Cappello, Zhichao Liang, James Samsom, Quanying Liu, He Lin, Christelle Baunez, Fang Liu, Ti‐Fei Yuan

**Affiliations:** ^1^ Shanghai Key Laboratory of Psychotic Disorders Brain Health Institute National Center for Mental Disorders Shanghai Mental Health Center Shanghai Jiaotong University School of Medicine and School of Psychology Shanghai 200030 China; ^2^ Co‐innovation Center of Neuroregeneration Nantong University Nantong Jiangsu 226019 China; ^3^ Department of Clinical Laboratory Diagnosis Changhai Hospital Shanghai 200433 China; ^4^ Institute of Mental Health and Drug Discovery, Oujiang Laboratory (Zhejiang Lab for Regenerative Medicine Vision and Brain Health), School of Mental Health Wenzhou Medical University Wenzhou Zhejiang 325000 China; ^5^ Institut de Neurosciences de la Timone UMR7289 CNRS & Aix‐Marseille Université Marseille France; ^6^ Shenzhen Key Laboratory of Smart Healthcare Engineering Department of Biomedical Engineering Southern University of Science and Technology Shenzhen 518055 China; ^7^ Campbell Family Mental Health Research Institute Centre for Addiction and Mental Health Toronto ON M5T1R8 Canada; ^8^ The Third Research Institute of Ministry of Public Security Shanghai 200438 China; ^9^ Department of Psychiatry, Physiology, Pharmacology and Toxicology and Institutes of Medical Science University of Toronto Toronto ON M5T 1R8 Canada

**Keywords:** addiction, deep brain stimulation, nucleus accumbens, prefrontal cortex

## Abstract

Deep brain stimulation (DBS) has emerged as a prospective treatment for psychiatric disorders; for example, DBS targeting the nucleus accumbens (NAc) abolishes addictive behaviors. However, neither the core pathway nor the cellular mechanisms underlying these therapeutic effects are known. Here, morphine‐induced conditioned place preference (CPP) in mice as an addiction model and NAc‐DBS combined with adeno‐associated virus gene delivery for activity‐dependent tagging, transgenic and chemogenetic manipulation of recruited neuronal networks are used. It is reported that a cortical‐accumbal pathway and local fibroblast growth factor 1 (FGF1) signaling in the medial prefrontal cortex (mPFC) are critical for NAc‐DBS to be effective in altering morphine CPP. It is shown that NAc‐DBS retrogradely activates mPFC neurons projecting to the NAc, and chemogenetic activation/inhibition of these DBS‐activated neuron ensembles in the mPFC reproduces the NAc‐DBS effects on CPP. Sustained therapeutic effects accompany reductions in local FGF1 binding to fibroblast growth factor receptor 1 (FGFR1) in these neurons. Additionally, overexpressing FGF1 in the mPFC‐NAc pathway abolishes the therapeutic effects of NAc‐DBS. These results demonstrate that the mPFC‐NAc pathway forms a top‐down motif to regulate the therapeutic effects of subcortical DBS on addiction. These results support the potential for addiction treatments involving FGF1 signaling and highlight the mPFC as a target for noninvasive brain stimulation.

## Graphical abstract

This research delineates that a cortical‐accumbal pathway and local FGF1 signaling in the medial prefrontal cortex are critical for NAc‐DBS to be effective in altering morphine CPP. These findings demonstrate a top‐down motif to regulate the therapeutic effects of subcortical DBS on addiction and support the potential for addiction treatments involving FGF1 signaling.

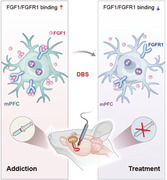

## 1 Introduction

Deep brain stimulation (DBS) is a therapeutic method in which constant electrical pulses are delivered to subcortical nuclei of the brain, notably the subthalamic nucleus (STN) in patients with Parkinson's disease and dystonia.^[^ ^1^, ^2^ ^]^ STN DBS has also been proposed as a therapeutic strategy to treat addiction,^[^ ^3^, ^4^, ^5^ ^]^ and efficacy was recently reported in one patient.^[^ ^6^ ^]^ The nucleus accumbens (NAc) has also been used as a target for DBS as a potential treatment for drug addiction in clinical patients.^[^ ^7^, ^8^ ^]^ Preclinical studies have demonstrated that NAc‐DBS could reduce drug‐adapted behaviors (e.g., conditioned place preference (CPP), locomotion sensitization), drug seeking, and consumption.^[^ ^9^, ^10^ ^]^ The core neural circuitry or cellular mechanism by which NAc‐DBS produces its effects, however, has not been fully investigated. Elucidating the neural substrate for NAc‐DBS is fundamental to optimizing the procedure for further therapeutic advances.

NAc‐DBS can activate different brain areas, including the local NAc, medial prefrontal cortex (mPFC), and amygdala.^[^ ^10^, ^11^ ^]^ Optogenetic‐mimicked DBS (i.e., long‐term depression of mPFC‐NAc projections) normalizes drug‐adapted synaptic changes and relevant addictive behaviors in mice,^[^ ^12^, ^13^ ^]^ highlighting the potential therapeutic importance of targeting cortical‐accumbal glutamatergic synapses or cortical neurons projecting to the NAc. Indeed, neuroimaging findings established the prefrontal cortical system as a critical network underlying many addictive behaviors, such as the generation of craving, impulsivity control, and decision‐making.^[^ ^14^, ^15^ ^]^ Identifying and harnessing key PFC‐centered circuit motifs and cellular ensembles underlying NAc‐DBS holds promise for developing noninvasive neuromodulation strategies to target pathway‐ and cell‐specific circuits for addiction treatment.

Here we discover the unexpected importance of the cortical‐subcortical motif underlying the therapeutic effects of NAc‐DBS against addiction. Electrical field simulation and active cell labeling revealed the recruitment of PFC‐NAc neuron ensembles in NAc‐DBS, while bidirectional chemogenetic exploration of the pathway proves its sufficiency and necessity. The sustained therapeutic effect accompanies a reduction in local fibroblast growth factor 1 (FGF1) binding to fibroblast growth factor receptor 1 (FGFR1) in these neurons; overexpressing FGF1 in the mPFC‐NAc pathway abolished the therapeutic effects of NAc‐DBS. Our results identify a prefrontal‐accumbal pathway and local FGF1 signaling in mPFC as a potential substrate for subcortical DBS therapy.

## 2 Results

### 2.1 NAc‐DBS Attenuates Morphine‐Induced CPP

**Figure 1:**
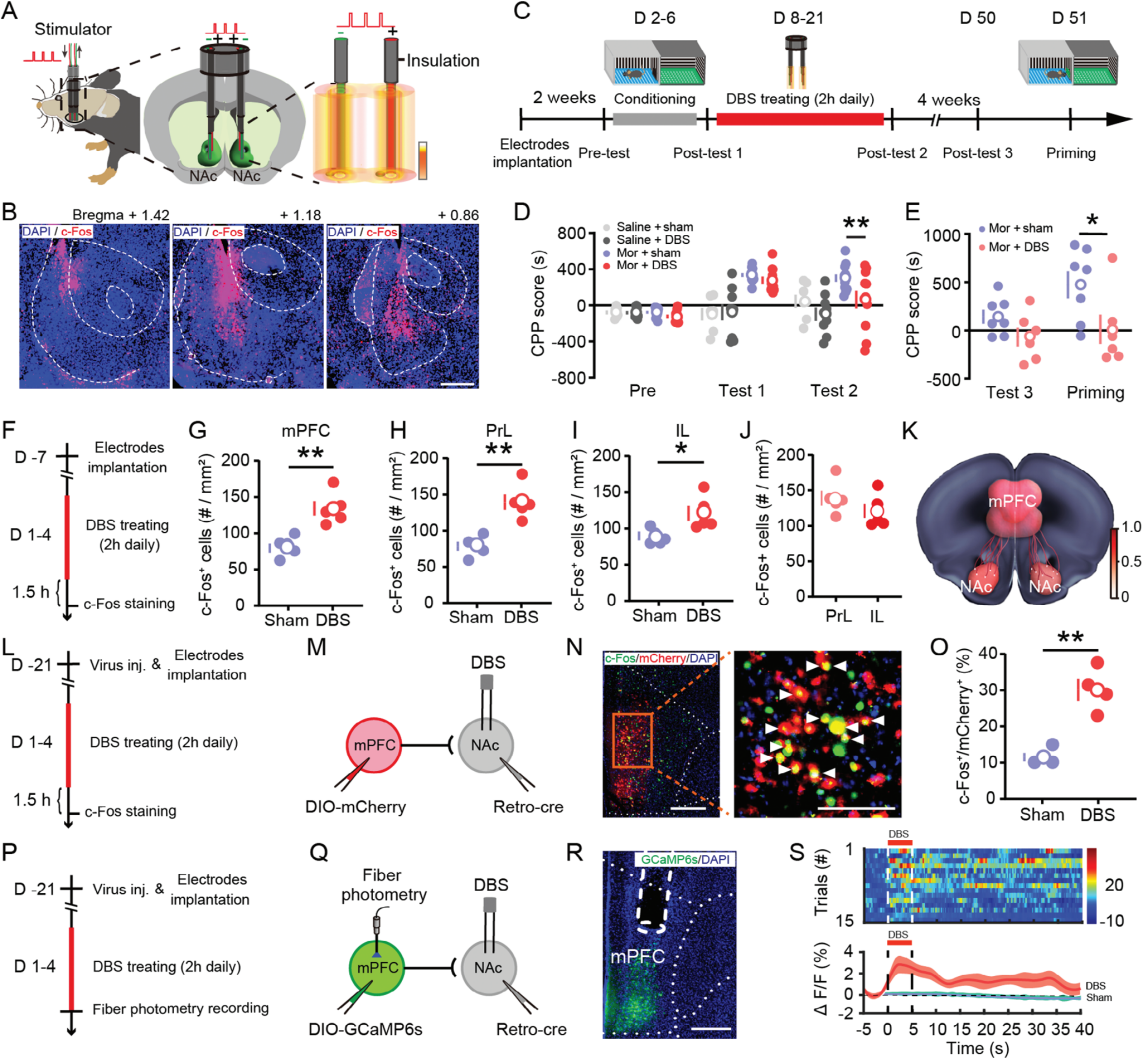
NAc‐DBS retrogradely activates prefrontal cortical neurons projecting to the NAc. A) Diagram of NAc‐DBS. B) Representative images of NAc‐DBS‐induced expression of c‐Fos in the NAc. Scale bar = 200 µm. C) Experimental timeline of NAc‐DBS treating morphine‐induced CPP. After 2 weeks of recovery from electrode implantation, mice received 5 days of morphine conditioning (15 mg kg^−1^, i.p.), then underwent 2 h NAc‐DBS for 14 constitutive days. CPP testing was performed either on day 22 or day 50/51. D) Summary bar graphs of CPP score in mice receiving morphine, NAc DBS, or control treatments. Two‐way ANOVA (treatment F_3,35_ = 12.32, *p* < 0.0001; test F_2,70_ = 16.65, *p* < 0.0001; interaction F_6,70_  =  6.716, *p* < 0.0001), Bonferroni corrected post hoc comparisons, saline‐sham, *n* =  7 mice; saline‐DBS, *n* =  9 mice; morphine‐sham, *n* =  12 mice; morphine‐DBS, *n* =  11 mice. E) Summary bar graphs of CPP score in the third and fourth post‐test for reinstatement of CPP. Two‐way ANOVA (treatment F_1,11_ = 5.120, *p* < 0.05; test F_1,11_ = 5.215, *p* < 0.05; interaction F_1,11_ = 2.119, *p* > 0.05), Bonferroni corrected post hoc comparisons; morphine‐sham, *n* =  7 mice; morphine‐DBS, *n* =  6 mice. F) Schematic of the experimental design. After a week of recovery from electrode implantation, mice received 2 h NAc‐DBS for 4 constitutive days, then were sacrificed after 1.5 h on the day 4. G–J) Quantification of the number of c‐Fos^+^ cells in the mPFC, PrL, and IL, assessed by an unpaired, two‐tailed Student's *t*‐test, *n* =  5 samples per group. K) A dynamical model relating neural activation and connectivity in the frontal cortex underlying NAc‐DBS. L) Schematic of the experimental design. M) Schematic of virus injection and electrode implantation. N) Representative images of expression of c‐Fos (green) in the mPFC‐NAc projection neurons (red) labeled by injection of Retro‐cre and DIO‐mCherry virus in DBS treated mice, merged c‐Fos‐positive mPFC‐NAc projection cells (yellow, indicated with white arrows, left). Scale bar = 200 µm. O) Quantification of the percentage of mPFC‐NAc projection neurons expressing c‐Fos; assessed by an unpaired, two‐tailed Student's *t*‐test; sham, *n* =  3 samples; DBS, *n* =  4 samples. P) Schematic of the experimental design. Q) Schematic of virus injection and electrode implantation. R) Representative images showing the expression of GCaMP6s and the placement of the fiber‐optic probe above the mPFC. Scale bar = 200 µm. S) Heatmap of fluorescence in mPFC‐NAc projection neurons in response to DBS (up), average delta F/F of fluorescence in mPFC‐NAc projection neurons in response to DBS and sham stimulation (down). Data represent mean ± SEM, ^*^ *p* < 0.05, ^**^ *p* < 0.01.

We first established the efficacy of NAc‐DBS on addiction‐related behavior in mice. Immunostaining of the neuronal activity marker, c‐Fos, shows that DBS resulted in the activation of neurons located in the NAc (**Figure** **1**A,B). NAc‐DBS did not impair recognition memory or locomotor activity, as measured using the novel object recognition test and open field test (Figure S1, Supporting Information). The timeline of morphine‐induced CPP and NAc‐DBS treatment is illustrated in Figure 1C. As expected, there were no differences in CPP scores in saline‐injected mice receiving either DBS or sham, while morphine‐injected mice with sham treatment showed significantly elevated CPP scores when compared to morphine‐injected DBS‐treated mice (Figure 1D). Notably, NAc‐DBS prevented morphine‐driven reinstatement assessed on day 51, after 4 weeks with no DBS treatment (Figure 1E), suggesting long‐term modulation of drug‐induced reward‐driven behavior.

### 2.2 NAc‐DBS Retrogradely Activates mPFC Neurons

We sought to understand the neuronal network activated by NAc‐DBS which could regulate addiction‐related behavior. Elevated numbers of c‐Fos^+^ cells were detected in the mPFC, in particular in both the prelimbic (PrL) and infralimbic (IL) cortex, after 4 days of 2 h/day NAc‐DBS (Figure 1F–I). This suggests neurons in the mPFC were activated during NAc‐DBS. To calculate the electrical field intensity and propagation, we simulated NAc‐seeded structural connections using a neural dynamical model, which indicated significant and direct electrical field propagation to mPFC by NAc‐DBS (Figure 1K; Figure S2, Supporting Information).

**Figure 2:**
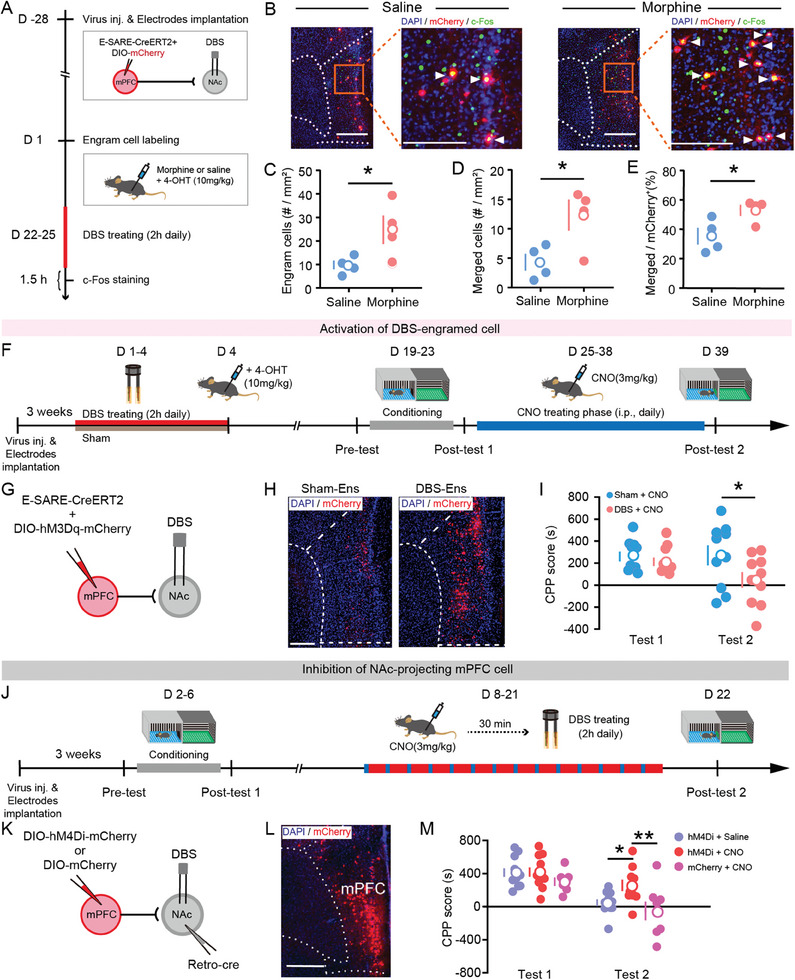
Activation of DBS‐recruited PFC neuronal ensembles mimics the effect of DBS on drug‐adapted behaviors. A) Schematic of the experimental design, virus injection, and electrode implantation. After 4 weeks of recovery, mice received morphine (15 mg kg^−1^, i.p.) or saline injection after 4‐OHT injection (10 mg kg^−1^, i.p.), then were subjected to 2 h NAc‐DBS for 4 constitutive days and sacrificed after 1.5 h on the day 25. B) Representative images of c‐Fos (green) and mCherry (red) fluorescence in saline and morphine engram cells. Scale bar = 200 µm. C–E) Quantification of the number of engram cells merged cells, and percentage of merged cells in engram cells in the mPFC in sham and DBS group mice, assessed by an unpaired, two‐tailed Student's *t*‐test; *n* =  4 samples per group. F) Schematic of the experimental design. After 3 weeks of recovery, mice received 2 h NAc‐DBS for 4 constitutive days and 4‐OHT injection (10 mg kg^−1^, i.p.). Then after the same experimental protocol of morphine exposure, mice were subject to CNO injection (3 mg kg^−1^, i.p.) daily for 14 consecutive days with CPP assessment on day 39. G) Schematic of virus injection and electrode implantation. H) Representative images of hM3Dq‐mCherry in the mPFC in sham and DBS‐treated mice. Scale bar = 100 µm. I) Quantification of morphine‐induced CPP score in sham and DBS‐treated mice. Two‐way ANOVA (treatment F_1,18_ = 2.002, *p* > 0.05; test F_1,18_ = 4.188, *p* > 0.05; interaction F_1,18_ = 2.080, *p* > 0.05), Bonferroni corrected post hoc comparisons, *n* =  10 mice per group. J) Schematic of the experimental design. K) Schematic of virus injection and electrode implantation. L) Representative images of expression hM4Di‐mCherry in the mPFC‐NAc projection neurons that were retrogradely labeled by injection of Retro‐cre virus. Scale bar = 300 µm. M) Quantification of morphine‐induced CPP score in hM4Di+saline, hM4Di+CNO, and mCherry+CNO group mice, saline or CNO (3 mg kg^−1^, i.p.) was injected 30 min before DBS. Two‐way ANOVA (treatment F_1,26_ = 29.44, *p* < 0.0001; test F_2,26_ = 3.950, *p* < 0.05; interaction F_2,26_ = 1.723, *p* > 0.05), Dunnett corrected post hoc comparisons, hM4Di+saline, *n* =  11 mice; hM4Di+CNO, *n* =  11 mice; mCherry+CNO, *n* =  7 mice. Data represent mean ± SEM, ^*^ *p* < 0.05, ^**^ *p* < 0.01.

We next investigated the circuit connection of NAc‐DBS‐activated mPFC neurons. rAAV2/2‐hSyn‐Retro‐Cre was injected into the NAc and AAV2/9‐hSyn‐DIO‐mCherry was injected into the mPFC to label mPFC‐NAc projecting neurons (Figure 1L,M). We found robust co‐expression of c‐Fos in retrograde‐labeled mPFC‐NAc projection neurons (Figure 1N,O). We further confirmed this phenomenon in live animals using calcium imaging taking advantage of the same retrograde labeling strategy (Figure 1P,Q). As seen in **Figure** **2**S, fiber photometry recording showed that NAc‐DBS triggered a rapid increase of GCaMP fluorescence in mPFC‐NAc projecting neurons, but not in sham conditions. Notably, the effect is accompanied by a local DA signal increase (Figure S3A–C, Supporting Information), while blocking D_1_ and D_2_ receptor signaling did not attenuate the activation of mPFC‐NAc neurons (Figure S3D,E, Supporting Information). This suggests that NAc‐DBS directly activates these mPFC‐NAc projections, possibly by retrograde activation.

### 2.3 Direct Activation of NAc‐DBS Neuron Ensembles Affects Morphine‐CPP

To confirm if the identified mPFC‐NAc projecting neuron ensemble was directly involved in addiction‐related behavior, we used an activity‐dependent neural tagging approach taking advantage of AAV2/9‐E‐SARECreERT2 to drive targeted recombination in activated neurons in the presence of 4‐hydroxytamoxifen (4‐OHT). We first labeled neurons activated during morphine treatment by injecting AAV2/9‐DIO‐mCherry into the mPFC and injecting morphine together with 4‐OHT, then performed 4 days of 2 hours/day DBS (Figure 2A). There was an increased density of mCherry‐labelled “engram” cells in the mPFC of morphine‐injected mice relative to saline‐injected controls (Figure 2B,C). There was also increased c‐Fos expression in labeled engram cells measured as both the number of co‐labeled cells and c‐Fos labeling as a percentage of mCherry labeling (Figure 2D,E). This suggests that mPFC neurons activated by morphine injection were the same ensemble as those activated by NAc‐DBS.

Next, we tried to manipulate the NAc‐DBS neuron ensemble using active labeling combined with a chemogenetic vector strategy. The NAc‐DBS procedure was performed in the presence of 4‐OHT to selectively induce excitatory hM3Dq‐receptor expression in DBS‐activated ensembles (Figure 2F–H). After 2 weeks, this population of neurons was then selectively activated daily with the hM3Dq agonist, clozapine‐N‐oxide (CNO), for 14 days following CPP conditioning. We observed a significant reduction in the CPP score of CNO‐treated animals relative to controls (Figure 2I). This suggests targeting the NAc‐DBS neuron ensemble in mPFC is sufficient to mimic the behavioral effects of DBS on CPP.

To further determine whether the mPFC‐NAc pathway serves as the necessary substrate for the treatments of NAc‐DBS, we injected rAAV2/2‐hSyn‐Retro‐Cre into the NAc and AAV2/9‐hSyn‐DIO‐hM4Di‐mCherry into the mPFC to drive expression of the inhibitory hM4Di‐receptor in mPFC‐NAc projecting neurons (Figure 2J–L). Mice were subjected to CPP conditioning and NAc‐DBS. AAV2/9‐DIO‐mCherry was used as a control to evaluate the effect of NAc‐DBS. CNO‐treated hM4Di‐tagged mice had significantly increased CPP scores relative to CNO‐treated mCherry‐tagged mice and hM4Di‐tagged mice without CNO (Figure 2M). These results show that inhibition of mPFC‐NAc projections abolishes the effects of NAc‐DBS on CPP; therefore, activation of these ensembles is necessary for NAc‐DBS to modulate addiction‐related behavior.

### 2.4 NAc‐DBS Evokes Molecular and Cellular Changes in mPFC Neurons

**Figure 3:**
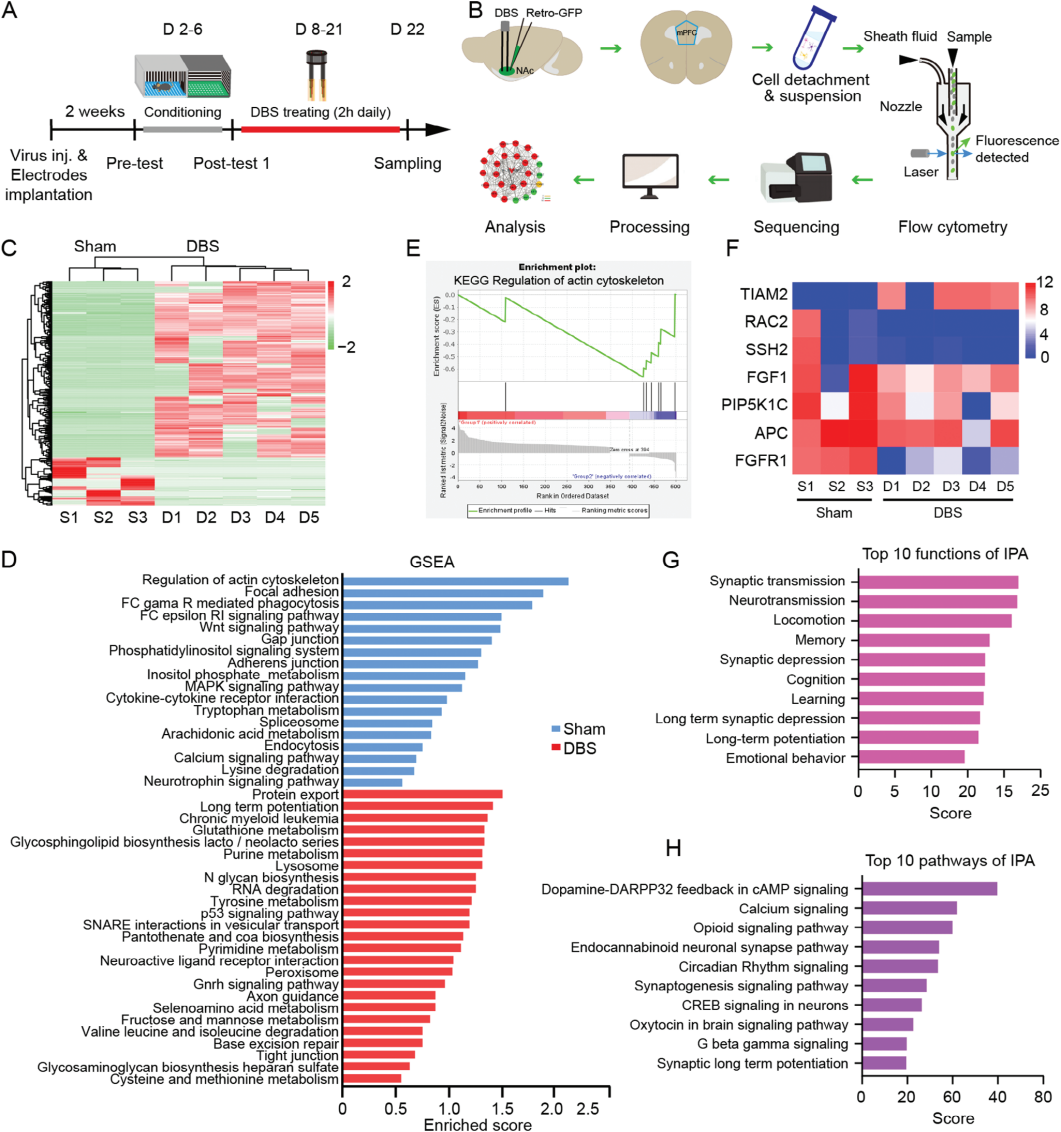
NAc‐DBS induced synaptic plasticity‐related transcriptomic changes in the mPFC‐NAc projection neurons. A) Schematic of the experimental design. B) Workflow for single‐cell sequencing of mPFC‐NAc neurons labeled by injection of Retro‐GFP virus in the NAc. C) Heatmap of differentially expressed genes in sham and DBS‐treated mice. Each column represents one mouse and each row represents one gene. Differences are ordered by hierarchical clustering. Color represents cell‐specific expression Z‐score. D) Gene set enrichment analysis of sham and DBS‐treated mice. E) Enrichment plot: Kyoto encyclopedia of genes and genomes (KEGG) regulation of actin cytoskeleton profile of the running enrichment score and positions of gene set members on the rank‐ordered list. F) Blue‐pink o' gram in the space of the analyzed gene sets in sham and DBS group mice. G) Ingenuity pathway analysis of top 10 functions. H) Ingenuity pathway analysis of top 10 canonical pathways.

To probe potential molecular changes during DBS treatment, we used single‐cell transcriptome sequencing of mPFC‐NAc projecting neurons identified by retrograde labeling with rAAV2/2‐hSyn‐Retro‐Cre‐GFP in mice subjected to morphine‐CPP and NAc‐DBS. GFP‐expressing mPFC‐NAc neurons were isolated with fluorescence‐activated cell sorting at day 22 (**Figure** **3**A,B). We detected 393 genes exhibiting up‐regulation in NAc‐DBS‐treated animals and 132 genes up‐regulation in sham controls (P value cutoff < 0.05) (Figure 3C). The most enriched gene set identified by gene set enrichment analysis (GSEA) was the regulation of the actin cytoskeleton (Figure 3D). Differentially expressed genes highlighted within the Kyoto Encyclopedia of Genes and Genomes (KEGG) analysis include: TIAM2, which was up‐regulated in NAc‐DBS treated mice; while RAC 2, SSH2, FGF1, PIP5KIC, APC, and FGFR1 which were up‐regulated in sham controls (Figure 3F). Ingenuity pathway analysis (IPA) revealed significant enrichment of genes involved in synaptic transmission, neurotransmission, locomotion, cognition, and in the various drug‐associated pathways, especially dopamine‐DARPP32 feedback in cAMP signaling, calcium signaling, and opioid signaling (Figure 3G,H). This analysis suggests the NAc‐DBS procedure may act through several pathways that regulate synaptic plasticity.

### 2.5 FGF1 Was Required for Treatment Effect of NAc‐DBS on Drug‐Evoked CPP

**Figure 4:**
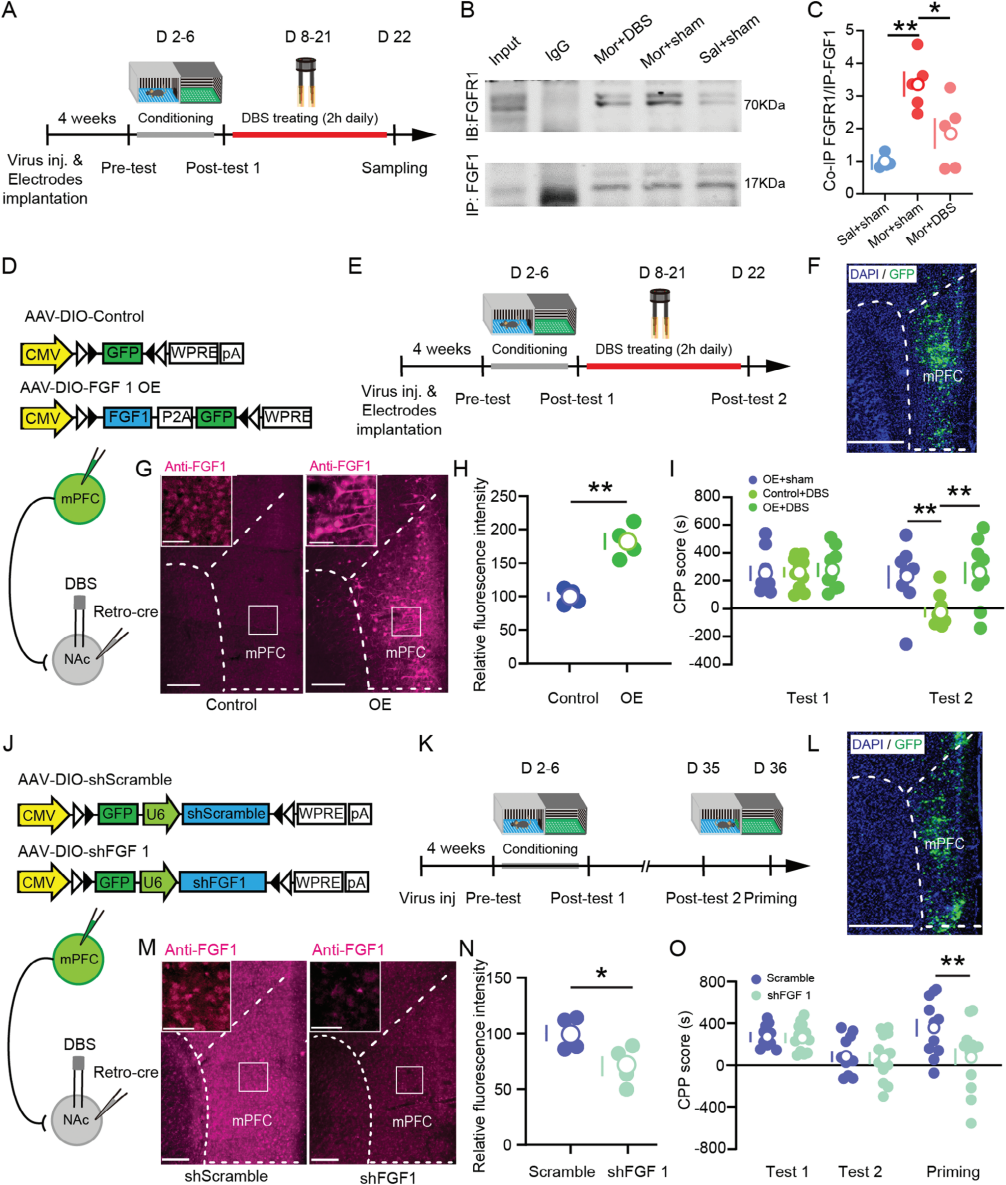
Overexpression of FGF1 in the mPFC‐NAc projection neurons prevents the effects of DBS on drug‐adapted behaviors. A) Schematic of the experimental design. B) Representative co‐immunoprecipitation (CoIP) images. In mouse brain lysate, FGF1 antibody, but not IgG (negative control), co‐immunoprecipitated with FGFR1. C) CoIP quantification. The level of FGF1‐FGFR1 complex increased in the morphine injection group as compared to levels in saline injection, reduced in NAc‐DBS treatment. Assessed by one‐way ANOVA (F_2,12_ = 11.88, *p* < 0.01), Tukey post hoc comparisons, *n* =  5 samples per group. D) Schematic of AAV vectors engineered to overexpress RNA targeting Fgf1 (AAV‐DIO‐FGF1 OE), control (AAV‐DIO‐Control) in mPFC‐NAc projection neurons. E) Schematic of the experimental design. (F) Representative images of expression FGF‐GFP in the mPFC‐NAc projection neurons labeled by injection of Retro‐cre virus into the NAc. Scale bar = 400µm. G) Representative immunohistochemistry images of FGF1 in OE and control mice. The region in the white box is shown at a higher resolution in the inset. Scale bar =  200 µm, 50 µm (inset). H) Immunohistochemistry showing upregulation of FGF1 expression by AAV‐DIO‐FGF1 OE. Unpaired, two‐tailed Student's *t*‐test, *n* =  4 samples per group. I) Quantification of morphine‐induced CPP score in OE+sham, control+DBS, and OE+DBS mice. Two‐way ANOVA (treatment F_1,24_ = 10.895, *p* < 0.001; test F_2,24_ = 2.947, *p* > 0.05; interaction F_2,24_ = 6.840, *p* < 0.001), Bonferroni corrected post hoc comparisons, OE+sham, *n* =  8 mice; control+DBS, *n* =  10 mice; OE+DBS, *n* =  9 mice. J) AAV vectors engineered to knockdown RNA targeting Fgf1 (AAV‐DIO‐shFGF1), and control (AAV‐DIO‐shScramble) in mPFC‐NAc projection neurons. K) Schematic of the experimental design. L) Representative images of expression of FGF‐GFP in mPFC‐NAc projection neurons labeled by injection of Retro‐cre virus into the NAc. Scale bar = 400 µm. M) Representative immunohistochemistry images of FGF1 in shRNA and control mice. The region in the white box is shown at a higher resolution in the inset. Scale bar =  200 µm, 50 µm (inset). N) Immunohistochemistry showing downregulation of FGF1 expression by AAV‐DIO‐shFGF1, unpaired, two‐tailed Student's *t*‐test, *n* =  4 samples per group. O) Quantification of morphine‐induced CPP score in Scramble and shRNA mice. Assessed by two‐way ANOVA (treatment F_1,23_ = 2.880, *p* > 0.05; test F_2,46_ = 6.719, *p* < 0.001; interaction F_2,46_ = 3.977, *p* < 0.05), Bonferroni corrected post hoc comparisons, Scramble, *n* =  11 mice; shFGF 1, *n* =  14 mice. Data represent mean ± SEM, ^*^ *p* < 0.05, ^**^ *p* < 0.01.

Among the differentially expressed actin cytoskeleton pathway genes found in the RNAseq analysis, FGF1 and FGFR1 were notable. FGF1 is an important member of fibroblast growth factors that have emerged as a key player in brain function and neuropsychiatric disorders. We used co‐immunoprecipitation to measure the binding of FGF1 to the FGFR1 in membrane extracts of mice receiving saline, morphine injections, or morphine with NAc‐DBS treatment (**Figure** **4**A). We found the ability of the FGF1 antibody to co‐immunopricipitate FGFR1 was significantly increased in morphine‐treated animals relative to controls, and NAc‐DBS treatment had reduced FGF1‐FGF1R binding compared to morphine‐treated animals (Figure 4B,C). There were no differences between groups in FGF1 direct immunoprecipitation. This shows there were no group differences in FGF1 levels, while FGF1 binding to FGFR1 was increased in the morphine‐treated group, suggesting that FGF1‐FGFR1 binding plays a role in the effects of morphine, and DBS could disrupt this interaction.

We next investigated the functional role of FGF1 within mPFC‐NAc projecting neurons in regulating NAc‐DBS effects on CPP. NAc rAAV2/2‐hSyn‐Retro‐Cre and mPFC AAV2/9‐CMV‐DIO‐FGF1‐GFP injections were used to over‐express FGF1 specifically in mPFC‐NAc neurons (AAV2/9‐DIO‐GFP as a control) (Figure 4D,F). The staining intensity of FGF1 protein in the mPFC was obviously enhanced in the AAV2/9‐CMV‐DIO‐FGF1‐GFP‐injected group (OE) as detected by immunohistochemistry (Figure 4G,H). NAc‐DBS significantly reduced CPP score relative to sham; while CPP score was not reduced in OE mice (Figure 4I). This shows that overexpression of FGF1 prevents NAc‐DBS effects on CPP, suggesting that a reduction in FGF1 is essential for modulating this effect. To further investigate this, we performed shRNA knockdown of FGF1 expression in mPFC‐NAc neurons using AAV2/9‐CMV‐DIO‐shFGF1‐GFP (Figure 4J,L). Immunohistochemistry showed a reduction in FGF1 staining in FGF1‐shRNA mice relative to scramble controls, suggesting successful knockdown (Figure 4M,N). Mice did not show differences in initial CPP; however, FGF1‐shRNA mice had significantly reduced CPP scores during morphine‐induced reinstatement compared to scramble controls (Figure 4O). This suggests that morphine induces CPP through increasing FGF1; therefore, knocking down FGF1 attenuated the conditioning. Together, this data shows that FGF1 signaling is important for regulating morphine‐induced CPP and the effects of NAc‐DBS on CPP.

## 3 Discussion

Our results revealed a direct PFC‐NAc pathway underlying the therapeutic effects of NAc‐DBS against addiction. We further identified FGF1‐FGFR1 binding as an essential molecular mechanism regulating this process. FGF1 overexpression abolishes NAc‐DBS effects, while FGF1 knockdown prevents reinstatement of drug behaviors. Together, the present study uncovers a dedicated top‐down structural motif along the cortical‐subcortical axis, presenting a proof of principle for using noninvasive brain stimulation (e.g., rTMS) targeting PFC to treat addictive disorders.

Currently, there are several hypotheses to explain the efficacy of DBS. DBS may cause the inhibition of local neurons via depolarization blockade or GABA release. In addition, it has also been proposed that the glial‐neuronal interactions play an important role in DBS efficacy.^[^ ^16^ ^]^ Retrograde activation, in which electrical stimulation from DBS can propagate backward up axons and passing fibers to activate wide brain networks, has been hypothesized as a mechanism to explain the modulation of distant targets by DBS, STN‐DBS in particular.^[^ ^17^, ^18^ ^]^ Indeed, although STN‐DBS tends to mostly mimic a functional inactivation of the STN^[^ ^4^, ^5^, ^19^ ^]^ it has been shown to increase global blood flow in many regions, including the stimulation site and caudate nucleus, as well as the primary sensorimotor and associative cortices.^[^ ^20^ ^]^ We show a focal increase in the expression of c‐Fos in regions within the mPFC suggestive of neuronal activation. In addition, we found abundant transcriptomic changes in mPFC‐NAc projecting neurons after NAc‐DBS. This supports a mechanism of retrograde activation of these projections, similar to the mechanism proposed for STN‐DBS activation of pedunculopontine nucleus‐STN and cortico‐STN circuits.^[^ ^17^, ^18^ ^]^ We cannot rule out reciprocal connections as a potential explanation for the activation of mPFC‐NAc projections, and our analysis focused on a specific population of projections from the mPFC. In the future, it will be important to elucidate the effects of NAc‐DBS on neuronal activation at the whole cortical scale, and potentially examine the possibility of reciprocal circuits.

The PFC is well known for its role in regulating addictive behaviors. Neuroimaging findings emphasize the important role of the PFC in cue‐induced reactivity and incubation of craving.^[^ ^14^ ^]^ Preclinical studies demonstrated that drug exposure results in long‐lasting strengthening of excitatory synaptic transmission from the mPFC to the NAc, while reversal of this synaptic plasticity resets adaptive behaviors.^[^ ^13^, ^21^ ^]^ Our findings further highlight PFC FGF1‐FGFR1 binding as an important signaling pathway in addiction. FGF1 was previously shown to regulate morphine sensitization when infused into the ventral tegmental area.^[^ ^22^ ^]^ Our results showed knockdown of FGF1 in mPFC‐NAc neurons abolishes morphine‐primed reinstatement of CPP (priming) while not affecting environmentally cued CPP (test 1 & 2), suggesting FGF1 in these projections was involved in encoding adaptive behavior caused by the drug itself rather than environmental cues. The impact of the knockdown of FGF1 in mPFC‐NAc neurons may be limited to this pathway. We found morphine elevated FGF1‐FGFR1 binding relative to controls, and NAc‐DBS counteracted morphine‐induced binding. FGF1 overexpression prevented NAc‐DBS effects on CPP. This is likely because elevated FGF1 levels increased the probability of transient FGF1‐FGFR1 binding which was beyond the ability of NAc‐DBS to correct. FGF1 and FGFR1 signaling could be exciting targets for addiction therapy. Additional research is needed to understand the specificity of this pathway and its effects on other addiction‐related models and drugs of abuse.

Numerous animal studies have investigated DBS for drug addiction therapy, with most reporting decreases in drug‐seeking behavior.^[^ ^5^, ^23^, ^24^ ^]^ However, stimulation effects are not consistent and depend on the timing, duration, and brain target of DBS. Creed et al. used a repeated cocaine exposure rodent model and found acute NAc‐DBS decreases cocaine‐induced behavioral sensitization, but this effect wears off 4–24 h after DBS.^[^ ^9^ ^]^ Other investigators reported that chronic or repeated DBS has long‐lasting effects on drug‐induced adaptive behavior in CPP and self‐administration models.^[^ ^4^, ^5^, ^23^, ^24^ ^]^ In our study, the effect of chronic NAc‐DBS on CPP score was persistent when testing 24 hours after DBS; therefore, we speculate that chronic DBS may have long‐lasting influences on behavioral sensitization.

This study has several limitations. First, we did not investigate the effects of manipulating DBS‐activated cortical ensembles on self‐administration behavior. Morphine‐induced CPP is only the first promising step toward generalization to addiction processes. Second, there were multiple cortical inputs to NAc medium spiny neurons (MSNs) that exhibit diversity in terms of plasticity, occurrence, and behavioral implications. Further refining the properties of the mPFC‐NAc connections that are recruited by DBS will greatly enhance our understanding of these circuits. Third, it will be important to further optimize the NAc‐DBS protocol; for example, by combining NAc‐DBS with PFC modulation, monitoring EEG signals in the PFC, or dissociating the NAc shell and core as in former studies.^[^ ^23^, ^25^ ^]^

In conclusion, our study reports a cortical‐subcortical motif underlying NAc‐DBS that is critical for beneficial effects in addiction‐related processes and investigates the molecular mechanisms that regulate this process. Our findings have important implications for the future of research into addiction therapies. Regions within the PFC are more accessible for noninvasive stimulation (e.g., transcranial magnetic stimulation). These strategies as well as targeting FGF1 and FGFR1 pathways hold potential for future research in addiction.

## 4 Experimental Section

### Animals

All experiments were approved by the Animal Experiment and Use Committee at the Shanghai Mental Health Center. Adult (6–8 weeks old) male C57BL/6 mice were used in this study. The animals were housed on a 12 h:12 h light‐dark cycle (lights on at 7 AM) with food and water provided ad libitum. The animals were randomly allocated to the experimental and control groups.

### DBS Electrodes

As previously described,^[^ ^26^ ^]^ the electrodes were composed of two platinum‐iridium wires coated with Teflon (777000, AM Systems). Platinum‐iridium wires were cut down to 8 mm and the coating was removed at the tips (the average distance between the two tips was 1 mm). Wires were paired and placed into stainless steel needles that were separated by a distance of 1.3 mm (62055, RWD) to form the electrodes. Wires were then soldered on an electric connector (132‐0377, RadioSpares) connecting to an electrical stimulation device (STG4008, Multichannel systems) by a slip ring (1196, Adafruit). The electrodes were deeply bound to the connector with epoxy resin.

### Surgery

For electrode implantation, all mice were anesthetized with 5% isoflurane and maintained at 1–2% on a stereotaxic apparatus (68 046, RWD). A small burr hole was made using a dental drill (78001, RWD), and the electrodes were bilaterally implanted and fixed to the skull using dental cement at the following coordinates: AP: +1.4 mm (from bregma); lateral: ±0.65 mm; dorsoventral: −4.2 mm (from the skull, according to Paxinos and Watson, 3rd edition) (Figure 1A). For fiber photometry recording, the fiber optic cannula (200 µm diameter; Newton Inc) was tilted to an angle of 6° and placed in the mPFC (AP: +1.95 mm; lateral: ±0.6 mm; dorsoventral: −2.0 mm), then fixed to the skull using dental cement. The scalp wound was closed with surgical sutures. After surgery, mice had 7–10 days of recovery.

Adeno‐associated virus (AAV) injection was performed with the same anesthetic and stereotaxic frame set‐up. Injections of the viral vector (200 nL) were performed using a compressed air delivery system. To specifically infect NAc‐projecting mPFC neurons with mCherry, GCaMP6s, hM4Di‐mCherry or eYFP, rAAV2/2 ‐hSyn‐Retro‐Cre (BrainVTA) was delivered bilaterally into the NAc (AP: +1.40 mm; lateral: ±0.65 mm; dorsoventral: −4.2 mm) of C57BL/6 mice, and AAV2/9 ‐hSyn‐DIO‐mCherry (BrainVTA), AAV2/9‐hSyn‐DIO‐GCaMP6s (BrainVTA), AAV2/9‐hSyn‐DIO‐hM4Di‐mCherry (BrainVTA) was injected into the mPFC (AP: +1.95 mm; lateral: ± 0.4 mm; dorsoventral: −1.8 mm). For engram cell labeling in the mPFC, 200 mL of a 1:1 volume mixture of AAV2/9‐E‐SARE‐CreERT2 (BrainVTA) and AAV2/9‐hSyn‐DIO‐mCherry (BrainVTA) or AAV2/9‐hSyn‐DIO‐hM3Dq‐ mCherry (BrainVTA) was injected into mPFC.

### NAc‐DBS

The following parameters were applied in the entire experiment for NAc‐DBS: frequency: 130 Hz, pulse width: 90 µs, and intensity: 50 µA. These were chosen according to a previous study^[^ ^9^ ^]^ and verified in preliminary experiments. To assess the effects of NAc‐DBS on morphine CPP, mice were randomly allocated to four experimental groups: saline‐sham, saline‐DBS, morphine‐sham, and morphine‐DBS. The morphine‐sham and morphine‐DBS groups were conditioned by morphine injection to generate place preference in the drug‐paired side with a standard CPP protocol; saline‐sham and saline‐DBS groups underwent the same procedure with saline injection. In the “spontaneous abstinence” phase, saline‐DBS and morphine‐DBS groups of mice were subjected to the DBS applied to the NAc 2 h daily in their home cage for 14 consecutive days, and CPP assessment was performed 24 h after stimulation (Figure 1C).

### Behavioral Paradigms

All behavioral tests were conducted during the light phase (7 AM–7 PM). Investigators were blinded to the experimental groups during the scoring.

### Conditioned Place Preference

As previously described,^[^ ^27^ ^]^ the CPP test was performed in a custom‐made two‐compartment CPP apparatus (42.5 cm long × 21.5 cm wide × 34 cm high) with distinct chambers (one with horizontal stripes walls and a grid floor, the other with vertical stripes walls and a floor with horizontal stripes). A video tracking system was used to record the trajectories of the mice. After 4 days for handling and habitation, on day 1 (pre‐test), the mice were allowed to freely explore both sides of the apparatus for 15 min to assess their base preference. Next, the conditioning phase was performed for 5 consecutive days. The mice received a morphine (15 mg kg^−1^, i.p.) injection in their nonpreferred side of the chamber and were left for 45 min before being returned to their home cage. Six hours later, the same mice received a saline injection in the opposite chamber and were left for 45 min before being returned to their home cage.

After conditioning, on day 7 (post‐test 1), the mice were allowed to explore both chambers for 15 min. The time spent in each chamber was analyzed. CPP score was calculated as the time spent in the saline‐paired chamber subtracted from the time spent in the morphine‐paired chamber. Mice that did not show a strong preference for the morphine‐paired chamber (<90 s) during post‐test 1 were excluded from further behavioral assays.^[^ ^28^ ^]^ The same 15 min procedure was used to assess CPP on day 22 after NAc‐DBS (post‐test 2).

### Morphine‐CPP Reinstatement Tests

For groups tested for reinstatement, a third 15 min post‐test was performed on day 50 (post‐test 3) using the CPP assessment procedure described above. To evaluate morphine‐induced reinstatement, mice were injected with a low dose of morphine (5 mg kg^−1^, i.p.), and after 30 min were placed in the CPP apparatus for another post‐test CPP assessment (day 51, post‐test 4 (priming)).

### Novel Object Recognition Test

A novel object recognition test was performed in an open field test chamber (40 cm × 40 cm × 40 cm) with 25 lux illuminance in the center.^[^ ^29^ ^]^ After 4 days of handling, all mice were transferred to the testing room and habituated for 30 min before the test. On the first day, the mice were placed in the chamber with two identical objects positioned on the diagonal and were allowed to explore the arena for 10 min. Twenty‐four hours later, the mice were placed in the chamber with one of the previous objects displaced and a novel object placed on the same diagonal and allowed to explore the arena for 10 min. The discrimination index was calculated as the time spent exploring the novel object divided by the total exploration time for both objects on the last day. The apparatus was cleaned with 75% ethanol between trials.

### Open Field Test

Motor activity was measured in an open field test chamber (40 cm × 40 cm × 40 cm) with 25 lux illuminance in the center. After 4 days of handling, all mice were transferred to the testing room and habituated for 30 min before the test. Mice were placed in the center of the chamber and allowed to explore the arena for 15 min. All activity was recorded with an infrared camera placed above the box. Locomotion and time spent in the center during the last 10 min were measured automatically by ANY‐maze software. The chamber was wiped with 75% ethanol after each test session.

### Immunohistochemistry

The timing of NAc‐DBS for each procedure was determined in preliminary experiments. It was found that NAc‐DBS induced a stable increase in c‐Fos expression in the mPFC after only 3 days of adaptive stimulation. Significant c‐Fos expression after 2 weeks of NAc‐DBS was also observed. To improve experimental efficiency, the 3‐day stimulation protocol in subsequent c‐Fos immunohistochemistry was opted to be applied and fiber‐optic recording experiments rather than using the full 2‐week protocol. Animals were euthanized by sodium pentobarbital overdose (50 mg kg^−1^, i.p.) 1.5 h after NAc‐DBS, then intracardially perfused with 0.01 m phosphate‐buffered saline (PBS) followed by 4% paraformaldehyde in 0.1 m phosphate buffer (PB). Brains were removed, post‐fixed overnight at 4 °C in PFA, then cryoprotected in 20% sucrose in 0.1 m PBS at 4 °C until they sank. Brains were sectioned (50 µm) on a freezing microtome (CM1950, Leica) in 4 series and were collected in PBS. Free‐floating sections containing the target brain areas were rinsed in PBS three times. Sections were incubated with 1:5000 diluted rabbit polyclonal anti‐c‐Fos antibody (156 002, SYSY) or 1:500 diluted rabbit polyclonal anti‐FGF1 antibody (PA5‐79249, Invitrogen) containing 3% normal donkey serum (v/v) and 0.25% Triton X‐100 (v/v) overnight at 4 °C. Sections were then washed with PBS three times and incubated with 1:1000 488 or 555‐donkey anti‐rabbit antibody (A‐21206, A‐31572, Invitrogen) containing 0.25% Triton X‐100 (v/v) for 2 h at 37 °C. Finally, sections were washed three times in PBS, mounted on slides, and coverslipped. Fluorescence images were collected using a confocal microscope (FV‐1000, Olympus). Digital images were processed using Olympus Fluoview Viewer 4.2b and Image J software to minimally adjust brightness and contrast.

### Quantification of c‐Fos and FGF1 Immunostaining

Immunohistological quantification was performed blinded to the treatment groups. The density of c‐Fos^+^ neurons was quantified using the optical dissector method. To quantify the percentage of NAc‐projecting mPFC neurons co‐labeled with c‐Fos, 7 mice received mPFC injections of AAV2/9‐hSyn‐DIO‐mCherry and NAc injection of rAAV2/2‐hSyn‐Retro‐Cre. The number of mCherry and c‐Fos double‐labeled neurons was counted in three consecutive brain sections (50 µm^−1^ section) within the mPFC. The percentage of mCherry/c‐Fos double‐labeled neurons was calculated as the percentage of the total number of mCherry/c‐Fos double‐labeled neurons relative to the total number of mCherry‐labeled neurons. The staining intensity of FGF1 protein in the mPFC was quantified by Image J software.

### Dynamical Model Relating Neural Activation and Connectivity

In‐silico experiments were conducted using a coarse‐grained linear dynamical system $\left(\dot{x}=Ax+u\right)$ among regions of interest (ROIs) in mPFC and NAc, where *x* characterizes the activation level of each ROI, *A*Is the strength of system‐level structural connections generated from the Allen Mouse Atlas (https://mouse.brain‐map.org/static/atlas), and *µ* is the neural stimulation signal in the NAc. The system state was randomly initialized and the neural dynamical system evolved until it reached a steady state, which was termed the level of activation after stimulation. As previously described,^[^ ^30^ ^]^ the system was stabilized to avoid infinite growth over time by normalizing structural connections as:

(1) $$A_{\mathrm{norm}}=\frac{A}{\lambda {\left(A\right)}_{\max}+c}\;-I$$

*I* denotes the identity matrix, λ(*A*)_max_ denotes the largest eigenvalue of the system, and *c* is the parameter that determines the rate of stabilization of the system. *c*  =  0.1 was set. The evolution of neural dynamics is shown in Figure S2B (Supporting Information).

### Fiber Photometry Recording

Fiber photometry was performed as previously reported.^[^ ^31^ ^]^ rAAV2/2‐hSyn‐Retro‐Cre was injected into the NAc and AAV2/9‐hSyn‐DIO ‐GCaMP6s (a calcium marker) was injected into the mPFC of mice. Before recording, mice received NAc‐DBS 2 h daily for 3 days, then they were placed in the fiber photometry system (Thinker Tech Nanjing Biotech). The laser power was adjusted at the tip of the optical fiber to 10–20 µW. After a stable fluorescence signal was obtained, 5 s DBS was delivered which was synchronously outputted to the fiber photometry system. Fluorescence values were obtained consecutively during the session. Each DBS trial was followed with a 10 min timeout. The DBS onset signal and fluorescence signal were recorded simultaneously by the fiber photometry system. Data were segmented based on DBS events within individual trials. Delta F/F 5 s before DBS onset was taken as the baseline. The fluorescence signal during the first 3 DBS trains was analyzed by investigators blinded to the experimental groups. Photometry data was analyzed with custom MATLAB code (MATLAB R2018b, MathWorks).

### Activity‐Dependent Neural Tagging Approach

To identify and manipulate mPFC neurons selectively recruited by morphine injection or NAc‐DBS, a viral genetic approach to permanently tag these cells with a fluorescent reporter or excitatory DREADD was employed. This strategy relied on expressing CreERT2 driven by the enhanced synaptic activity‐responsive element (E‐SARE). Recombination of target alleles following induction of E‐SARECreERT2 depends on the presence of the estrogen receptor ligand 4‐hydroxytamoxifen (4‐OHT, H6278, Sigma–Aldrich), which restricts neuronal tagging to a period of several hours following activity.^[^ ^32^ ^]^ To label neurons active during morphine injection, a 1:1 volume mixture of AAV2/9‐E‐SARECreERT2 and AAV2/9‐DIO‐mCherry was bilaterally injected into mPFC to label mPFC neuronal ensembles activated by saline or morphine exposure. To manipulate neurons active during NAc‐DBS, a 1:1 volume mixture of AAV2/9‐E‐SARECreERT2 and AAV2/9‐DIO‐hM3Dq‐mCherry was bilaterally injected into the mPFC. To open a labeling window, mice received an injection of 4‐OHT (10 mg kg^−1^, i.p.) after either saline or morphine exposure or NAc‐DBS. 4‐OHT was formulated as previously described.^[^ ^33^ ^]^ In brief, 4‐OHT was dissolved in DMSO (40 mg mL^−1^) and further mixed in sterile saline containing 2% TWEEN 80, resulting in a 1 mg mL^−1^ 4‐OHT solution.

### Chemogenetic Experiments

To specifically inhibit NAc‐projecting mPFC neurons, rAAV2/2‐hSyn‐Retro‐Cre was bilaterally injected into the NAc and AAV2/9‐hSyn‐Dio‐hM4Di‐mCherry into the mPFC to achieve selective expression of designer receptors (DREADD; hM4Di) (AAV2/9‐DIO‐hM4Di‐mCherry or AAV2/9‐DIO‐mCherry as a control). After full viral expression and CPP conditioning were achieved, mice received i.p. injections of vehicle or clozapine N‐oxide (CNO, 3 mg kg^−1^, C2041, LKT) 30 min before DBS onset for 14 consecutive days and conducted CPP test 24 h after stimulation.

### RNA‐Seq Analysis

NAc‐projecting mPFC neurons were labeled by NAc injection of rAAV2/2 ‐hSyn‐Retro‐GFP (Brain VTA). After CPP conditioning and DBS treatment, animals were sacrificed, their brains were removed, and the mPFC was precisely isolated and processed into a single‐cell suspension according to a previously published method.^[^ ^34^ ^]^ Briefly, mice were anesthetized with isoflurane (3.5%) and decapitated. Brains were rapidly extracted and immersed in cold artificial cerebrospinal fluid (ACSF) for 5 min. The brain was then sectioned into 300 µm slices using a vibratome. The mPFC was isolated and digested in Hibernate‐A medium containing 10 U mL^−1^ Papain and 100 U mL^−1^ DNase I at room temperature for 5 min with gentle trituration. The resulting suspension was filtered through a 75 µm mesh and purified using a Percoll gradient. Finally, the cells were resuspended in Hibernate‐A medium supplemented with 0.04% BSA and 1–5 mm EDTA and maintained on ice. A cell counting plate was used to ensure that the final concentration was within 1 × 10^6^–1 × 10^7^ cells mL^−1^. GFP+ cells were separated with fluorescence‐activated cell sorting (BD FACSARIA III). The sorted cells were transferred in Hibernate‐A medium with 0.2% BSA. The percentage of GFP^+^ cells in all sorted cells was checked to ensure it was >90%. Sorted cells were transferred to a cell lysis buffer containing barcoded reverse transcription primers. Reverse transcription and amplification were performed using SMART‐Seq HT Kits according to the manufacturer's instructions. The cDNA concentration was determined using Qubit and length distribution was measured with an Agilent 2100 Bioanalyzer. Sequencing was performed using an Illumina NovaSeq6000, giving ≈60 m raw reads per mouse and the proportion of bases with a mass greater than 20 (Q20) in each direction is not less than 85% after de‐multiplexing. Raw sequence data were filtered using Seqtk with Hisat2 for genome mapping (version: 2.0.4) and then aligned to the mouse genome (GRCm38.p4 (mm10)) with STAR (version 2.7.0a). Analysis of differential expression was performed using the edgeR R package (3.12.1). Differentially expressed genes were identified by a corrected *p* value < 0.05. Kyoto Encyclopedia of Genes and Genomes (KEGG) and GSEA were implemented using the cluster Profiler R package. The hierarchical clustering heat map was generated with ggplot.

### Co‐Immunoprecipitation and Western Blot

All membrane protein samples from mice were homogenized using the Minute Plasma Membrane Protein Isolation and Cell Fractionation Kit (Invent, SM‐005). For co‐immunoprecipitation experiments, 500 µg solubilized membrane protein extracted from mouse brain tissue was incubated in the presence of protein A/G plus agarose (Santa Cruz Biotechnology) for 1 h at 4 °C followed by the addition of primary antibody or control IgG antibody (2 µg) with freshly washed protein A/G plus agarose (25 µL) overnight at 4 °C. Pellets were washed, boiled for 8 min in SDS sample buffer, and subjected to SDS‐PAGE. Total protein extract (60–100 µg) was used as a control in each experiment. After the transfer of proteins into nitrocellulose, membranes were subjected to Western blot. Protein levels were quantified by densitometry (software: Image J, NIH). Antibodies used were: anti‐Flg/FGFR1 (1:200, Santa Cruz Biotechnology, mouse), anti‐ Acidic FGF/FGF1 (1:1000, Cell Signaling Technology, rabbit), anti‐FGF Receptor 1 (1:1000, Cell Signaling Technology, rabbit), and IRDye Secondary Antibodies (1:25 000, LI‐COR).

### AAV‐Mediated Gene Transfer Approach

To specifically manipulate FGF 1 in NAc‐projecting mPFC neurons, rAAV2/2‐hSyn‐Retro‐Cre was bilaterally injected into the NAc and AAV2/9‐CMV‐DIO‐FGF1‐EGFP or AAV2/9‐CMV‐DIO‐shFGF1‐GFP into the mPFC to achieve over‐express or down‐regulate FGF1 specifically in mPFC‐NAc neurons (AAV2/9‐DIO‐GFP as a control). Recombinant viruses were generated by Brain VTA. For the over‐expression of the FGF1 experiment, after full viral expression and CPP conditioning were achieved, mice received DBS treatment for 14 consecutive days and conducted a CPP test 24 h after stimulation. For the knocked‐down FGF1 experiment, after full viral expression and CPP conditioning were achieved, mice received 28 days of spontaneous abstinence, and the CPP test was conducted 24 h after stimulation and the morphine‐CPP reinstatement test 24 h after the CPP test.

### Statistics

All statistical analyses were performed using SPSS 18.0 and GraphPad Prism 7 software. The Shapiro–Wilk test was used to check the normality of data. Unpaired Student's *t*‐tests were used to compare treatment groups receiving active or sham DBS for immunohistochemistry analysis. Two‐way ANOVAs were used to analyze CPP data with treatment (sham vs DBS) as a between‐group variable and post‐test (Test 1, Test 2, where appropriate Test 3, and Priming) as a within‐group variable, followed by Bonferroni post hoc tests for pairwise comparisons between groups. Coimmunoprecipitation data were analyzed using a one‐way ANOVA to compare treatment groups (saline+sham, morphine+sham, morphine+DBS) followed by post hoc Tukey's test. Statistical details are reported in the figure legends, including sample size, *p* values, and the types of statistical tests performed. Data analysis was performed by experimenters blinded to the experimental groups. Statistical significance was set at *p* < 0.05.

## Conflict of Interest

The authors declare no conflict of interest.

## Author Contributions

W.‐K. G., X. L. and L. W. equally contributed to this work. W.K.G., X.L., L.W., C.B., F.L., and T.F.Y. conceived and designed the study. W.K.G., X.L., L.W., Q.Y., A.T.C., Z.L., J.S., Q.L., and H.L. collected the data. W.K.G., X.L., L.W., Q.Y., A.T.C., Z.L., J.S., Q.L., H.L., C.B., F.L., and T.F.Y. analyzed the data and wrote the manuscript together.

## Supporting information



Supporting Information

## Data Availability

The data that support the findings of this study are available on request from the corresponding author. The data are not publicly available due to privacy or ethical restrictions.
