# Supplementary material for: Prefrontal FGF1 Signaling is Required for Accumbal Deep Brain Stimulation Treatment of Addiction
Source: Adv Sci (Weinh). 2025 Feb 27;12(16):2413370. doi: 10.1002/advs.202413370 (PMC12021060; doi:10.1002/advs.202413370)
Supplement: Supplementary file 1 — Supporting Information [file ADVS-12-2413370-s001.docx]

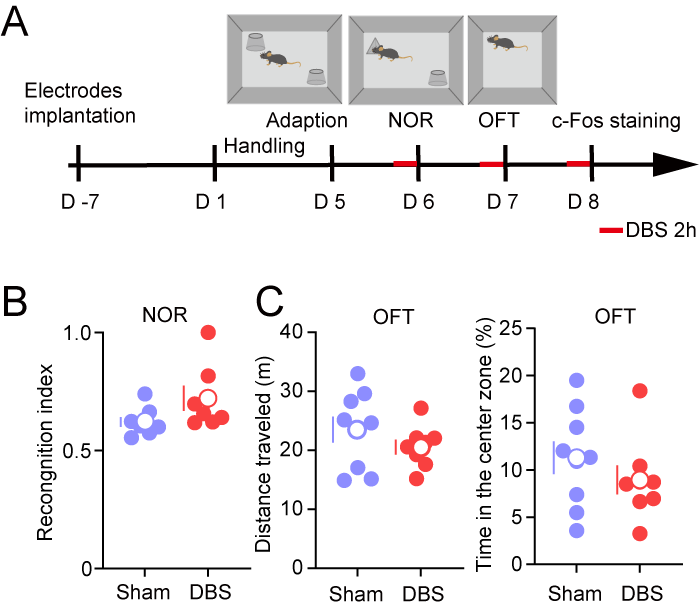


Supplementary Fig. 1 **NAc-DBS did not affect cognition or locomotor activity**.

(A) Schematic of the experimental design. After a week recovery from electrode implantation and 4 days of handling, mice were subjected to the novel object recognition (NOR) adaption phase and 24 hours later underwent 2 h NAc-DBS before NOR testing, followed 24 hours later by open field test (OFT).

(B)(C) Effects of NAc-DBS on NOR test and OFT, compared with sham group, unpaired, two-tailed Student’s t test; sham, n = 9 mice; DBS, n = 8 mice.

Data represent mean ± SEM.


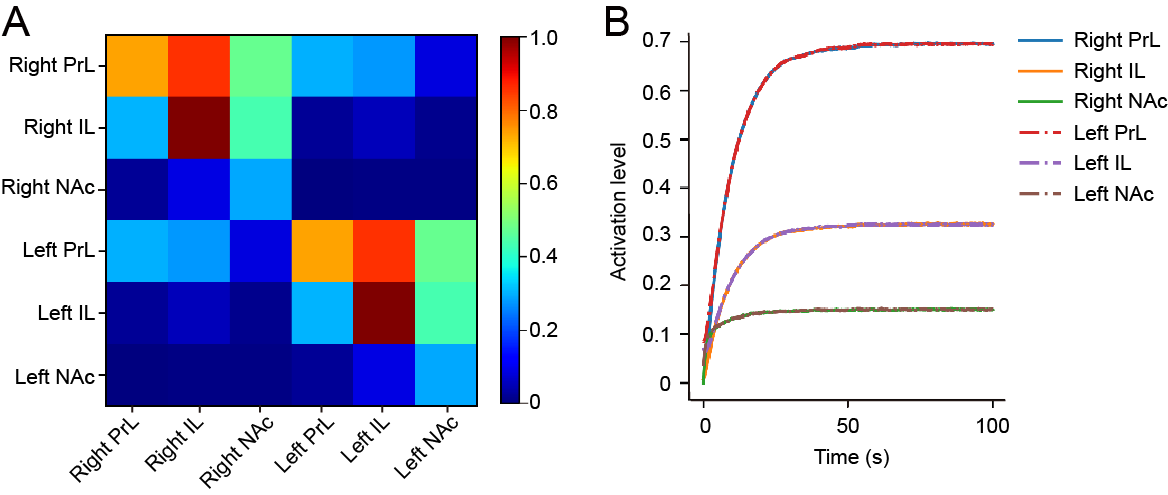


Supplementary Fig. 2. **NAc-DBS increased activation level in the mPFC in a mouse network activity model.**

1. Structural connections among NAc, PrL and IL.
2. Evolution of neural dynamics after stimulation of the NAc.


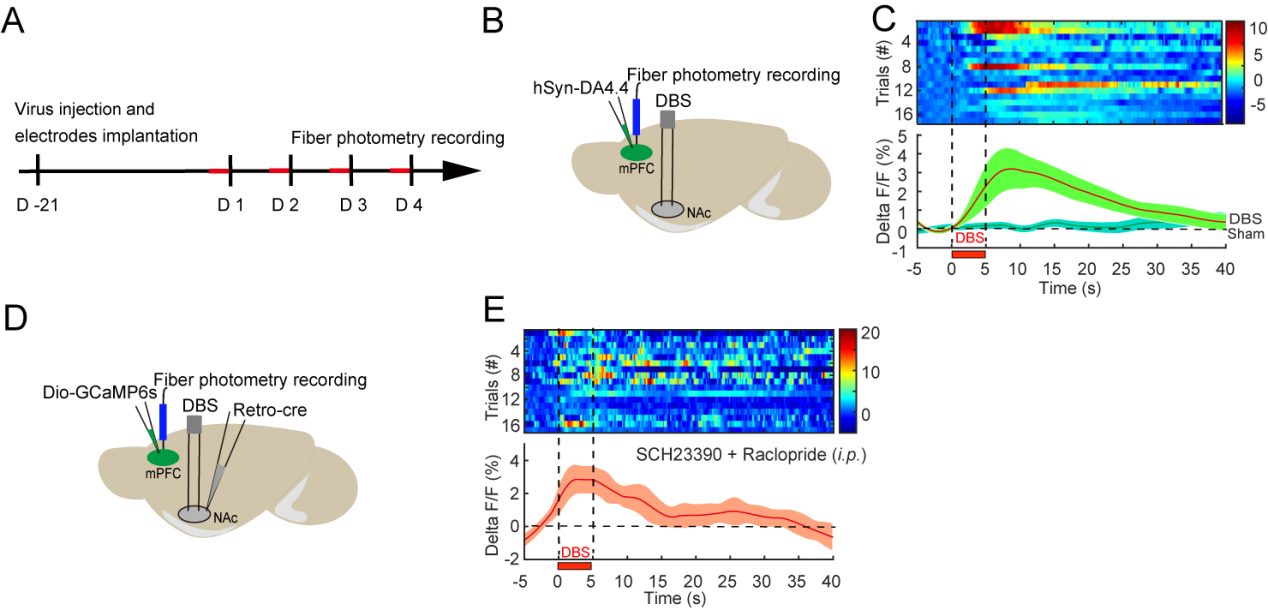


Supplementary Fig. 3 **NAc-DBS activated mPFC-NAc projecting neurons independent of the local dopamine (DA) signal**.

1. Schematic of virus injection and electrode and fiber implantation.
2. Schematic of DA sensors, virus injection, and electrode and fiber implantation. After 3 weeks of recovery, mice received 2 h NAc-DBS for 4 consecutive days, with fiber photometry recordings on day 4. Mice were subjected to 5s NAc-DBS with simultaneous DA sensor recordings.
3. Heatmap of DA sensor fluorescence in the mPFC in response to DBS (up), average delta F/F of fluorescence in the mPFC in response to DBS and sham stimulation (down).
4. Schematic of GCaMP6s virus injection, and electrode and fiber implantation. After 3 weeks of recovery, mice received 2 h NAc-DBS for 4 consecutive days, and fiber photometry recordings were done on day 4. Mice were injected with SCH23390 (D1R antagonist, 0.5mg/kg, i.p.) and Raclopride (D2R antagonist, 2mg/kg, i.p.), and 30 min later mice received 5s NAc-DBS with simultaneous GCaMP6s signal recordings.
5. Heatmap of fluorescence in mPFC-NAc projections neurons in response to DBS (up), average delta F/F of fluorescence in the mPFC-NAc projection neurons in response to DBS (down) with DA antagonists.


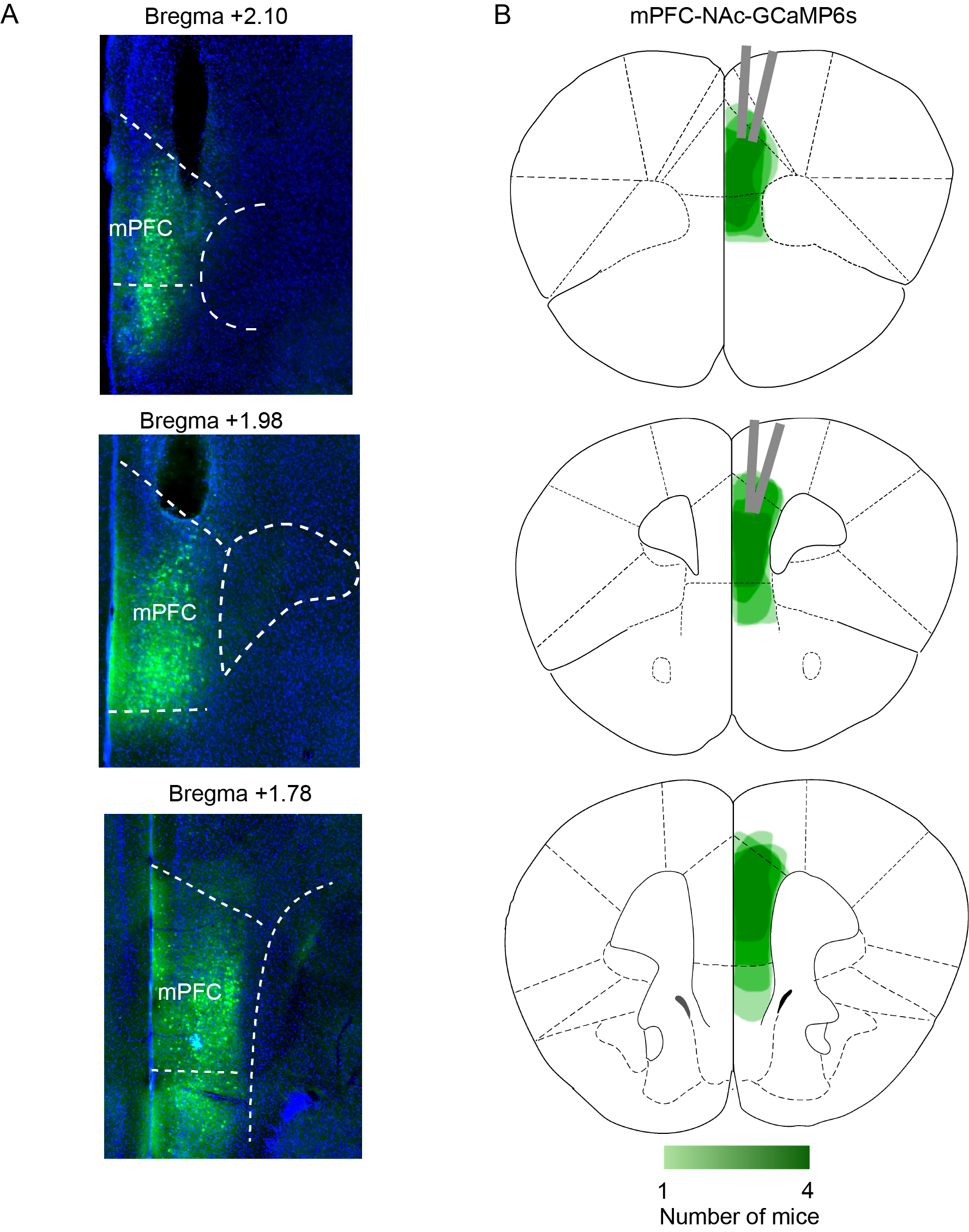


Supplementary Fig. 4 **Viral targeting and fiber placement in dmPFC for fiber photometry recording**.

1. Anterior to posterior brain slices from a mouse with the expression of GCaMP6s and the placement of fiber-optic probe above the mPFC.
2. Overlay of GCaMP6s expression areas in 4 mice which received NAc-DBS. Intensity of green color is proportional to the number of mice expressing virus in the marked area


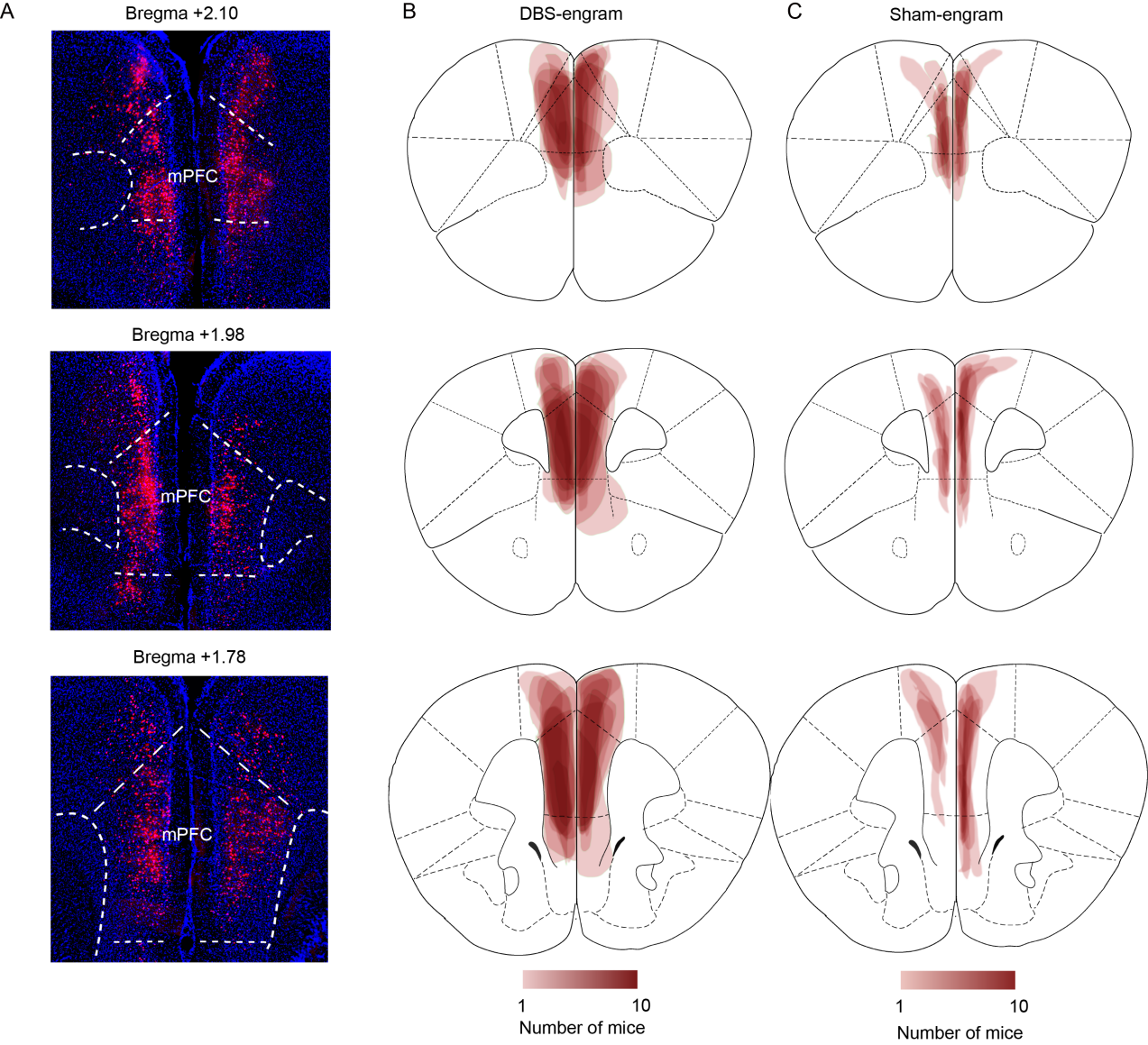


Supplementary Fig. 5 **Virus expression of experiments, related to Figure 2I**.

1. Anterior to posterior brain slices from a mouse with the expression of hM4Di-mCherry into mPFC.
2. (C) Overlay of DBS engram cell-mCherry (B), sham engram cell-mCherry (C) expression in mice. Intensity of red color is proportional to the number of mice expressing virus in the marked area.


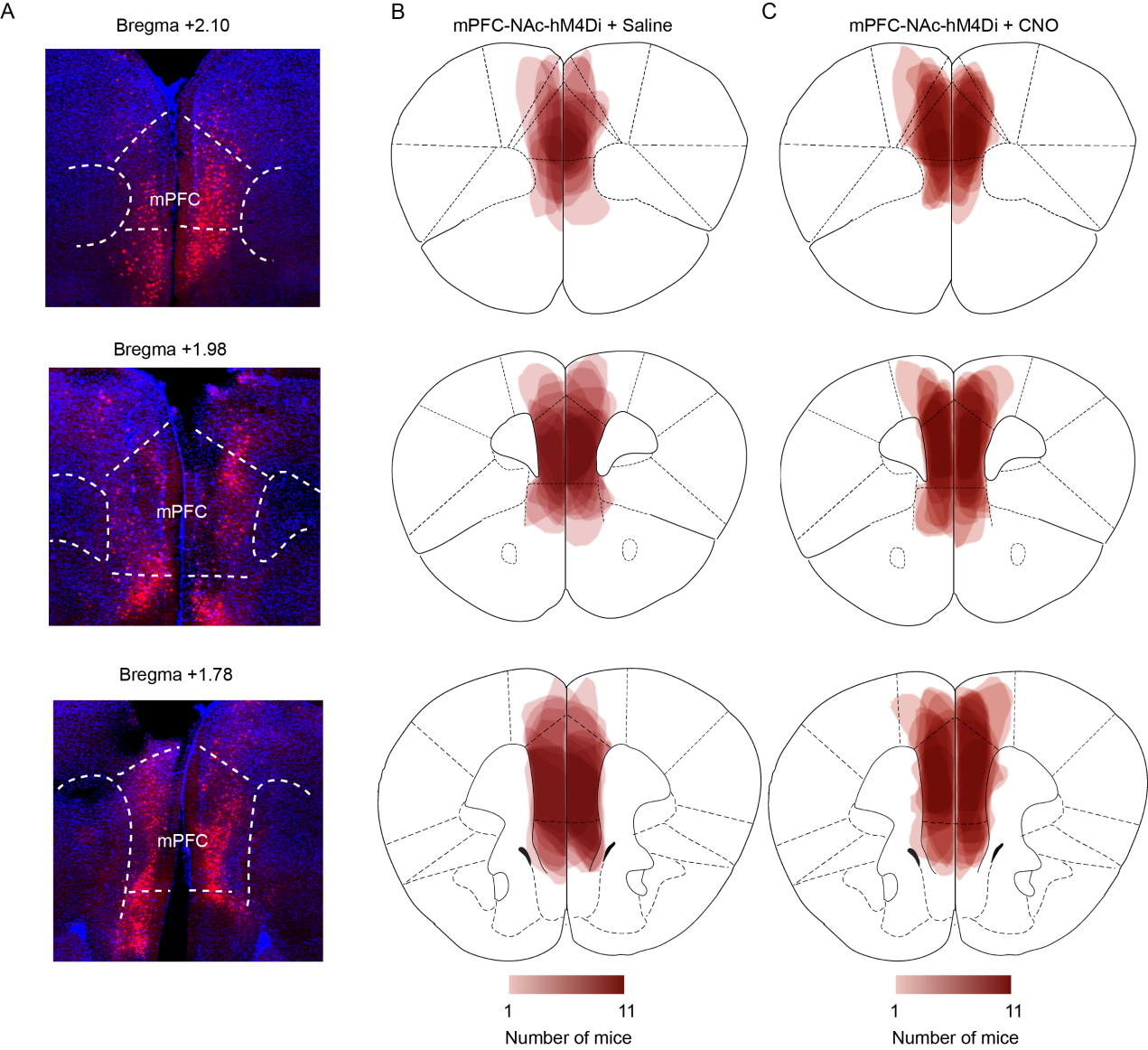


Supplementary Fig. 6 **Virus expression of experiments, related to Figure 2M**.

1. Anterior to posterior brain slices from a mouse with the expression of hM4Di-mCherry into mPFC.
2. (C) Overlay of hM4Di-mCherry expression in mice. Intensity of red color is proportional to the number of mice expressing virus in the marked area.


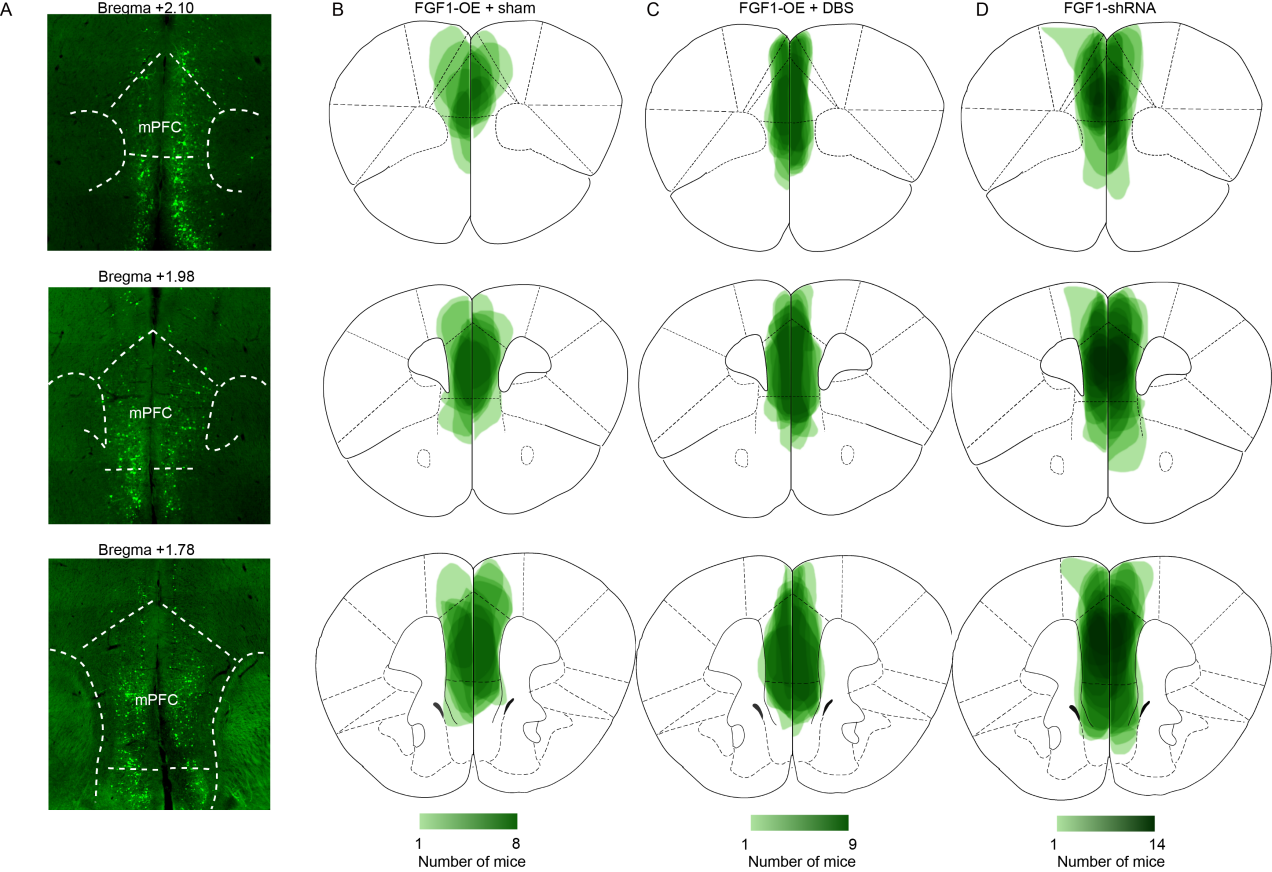


Supplementary Fig. 7 **Virus expression of experiments, related to Figure 4**.

1. Anterior to posterior brain slices from a mouse with the expression of FGF1-OE-GFP into mPFC.
2. (C) (D) Overlay of FGF1-OE-GFP (B,C) and FGF1-shRNA-GFP (D) expression in mice. Intensity of green color is proportional to the number of mice expressing virus in the marked area.


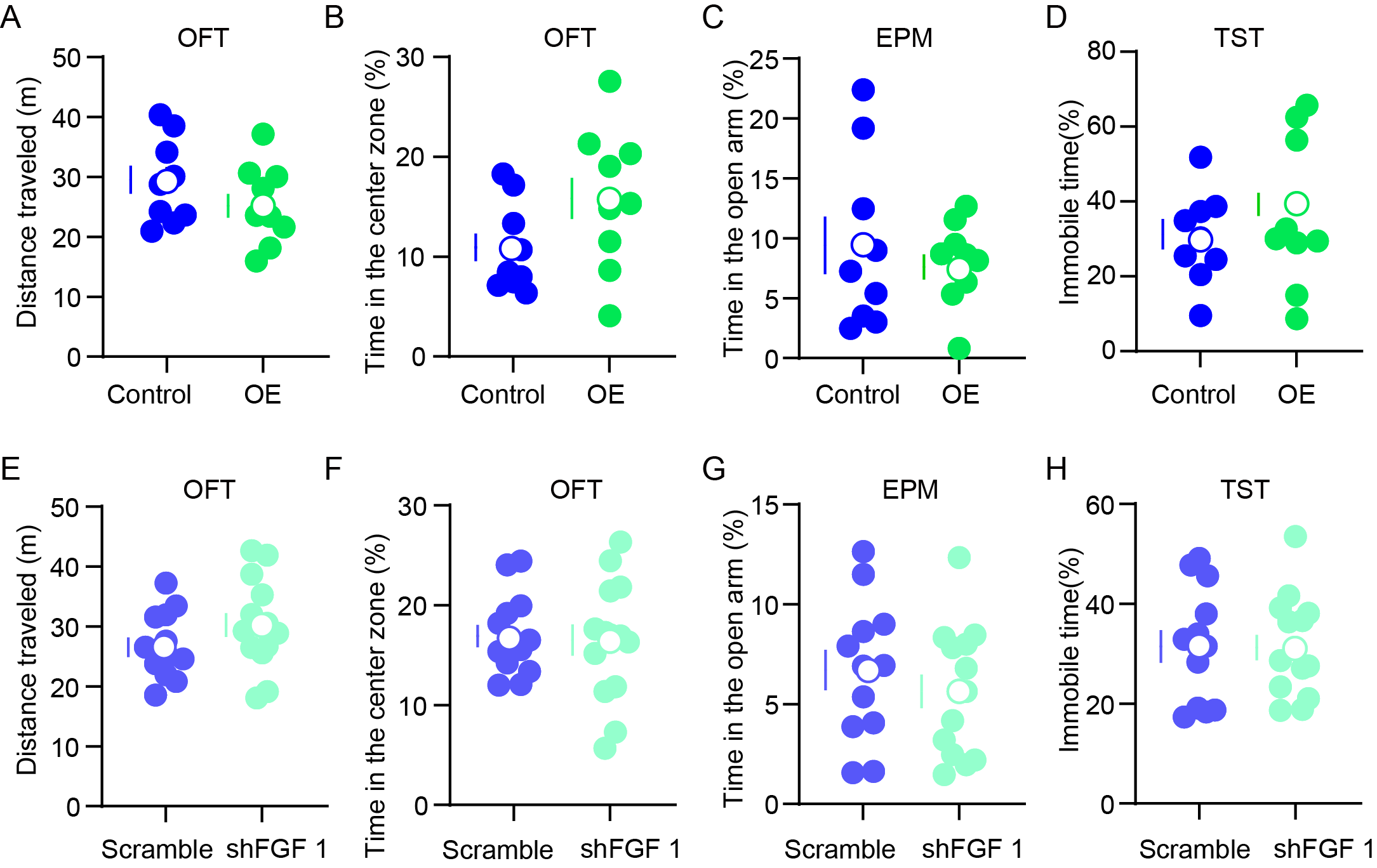
Supplementary Fig. 8 **Manipulation of FGF1 did not affect behaviour in mice**.

1. (B)(C)(D) Effects of overexpression of FGF1 on open field test (OFT) for locomotor activity, elevated plus maze (EPM) for anxiety-like behaviour, and tail-suspension test (TST) for depression-related behaviour, compared to control, unpaired, two-tailed Student’s t test; control, n = 9 mice; OE, n = 9 mice.

(E)(F)(G)(H) Effects of FGF1 knockdown on OFT, EPM and TST, compared with scramble shRNA control, unpaired, two-tailed Student’s t test; Scramble, n = 11 mice; shFGF 1, n = 14 mice.

Data represent mean ± SEM.

**Table S1.**

| **Figure number** | **Groups** | **Statistical results** |
| --- | --- | --- |
| 1D | saline-sham, n = 7 mice saline-DBS, n = 9 mice morphine-sham, n = 12 mice  morphine-DBS, n = 11 mice | Two-way Repeated Measures ANOVA  Treatment: F_3, 35_ = 12.317, P = 0.000012  Test: F_2, 70_ = 16.648, P = 0.000001  Interaction: F_6,70_ = 6.717, P = 0.000012  Bonferroni corrected post hoc comparisons  sham+mor vs. DBS+mor  Pre: >0.9999  Test 1: >0.9999  Test 2: 0.0069 |
| 1E | morphine-sham, n = 7 mice  morphine-DBS, n = 6 mice | Two-way ANOVA  Treatment: F_1, 11_ = 5.120, P = 0.0449  Test: F_1, 11_ = 5.215, P = 0.0433  Interaction: F_1, 11_ = 2.119, P = 0.1734  Bonferroni corrected post hoc comparisons  sham+mor vs. DBS+mor  Context: 0.4895  Priming: 0.0257 |
| 1G | sham, n = 5 samples  DBS, n = 5 | Unpaired t-test, two-tailed  t_8_ = 4.460, P = 0.0021 |
| 1H | sham, n = 5 samples  DBS, n = 5 samples | Unpaired t-test, two-tailed  t_8_ = 4.918, P = 0.0012 |
| 1I | sham, n = 5 samples  DBS, n = 5 samples | Unpaired t-test, two-tailed  t_8_ = 2.850, P = 0.0215 |
| 1J | PrL, n = 5 samples  IL, n = 5 samples | Unpaired t-test, two-tailed  t_8_ = 1.269, P = 0.2401 |
| 1O | sham, n = 4 samples  DBS, n = 4 samples | Unpaired t-test, two-tailed  t_6_ = 5.733, P = 0.0012 |
| 2C | saline, n = 4 samples  morphine, n = 4 samples | Unpaired t-test, two-tailed  t_6_ = 2.626, P = 0.0393 |
| 2D | saline, n = 4 samples  morphine, n = 4 samples | Unpaired t-test, two-tailed  t_6_ = 2.631, P = 0.0390 |
| 2E | saline, n = 4 samples  morphine, n = 4 samples | Unpaired t-test, two-tailed  t_6_ = 2.616, P = 0.0398 |
| 2I | sham+CNO, n = 10 mice  DBS+CNO, n = 10 mice | Two-way ANOVA  Treatment: F_1, 18_ = 2.002, P = 0.1742  Test: F_1, 18_ = 4.188, P = 0.0556  Interaction: F_1, 18_ = 2.080, P = 0.1664  Bonferroni corrected post hoc comparisons  sham+CNO vs. DBS+CNO  Test 1: >0.9999  Test 2: 0.0368 |

| 2M | hM4Di+saline, n = 11 mice  hM4Di+CNO, n = 11 mice  mCherry+CNO, n = 7 mice | Two-way Repeated Measures ANOVA  Treatment: F_1, 26_ = 29.44, P < 0.0001  Test: F_2, 26_ = 3.950, P=0.0318  Interaction: F_2, 26_ = 1.723, P = 0.1984  Dunnett multiple comparisons  hM4Di+saline vs. hM4Di+CNO  Test 1: 0.9992  Test 2: 0.0457  hM4Di+CNO vs. mCherry+CNO  Test 1: 0.4939  Test 2: 0.0092 |
| --- | --- | --- |
| 4C | sal+sham, n = 5 samples  mor+sham, n = 5 samples  mor+DBS , n = 5 samples | One-way ANOVA  F_2, 12_ = 11.883, P = 0.0014  Tukey corrected post hoc comparisons  sal+sham vs. mor+sham: P = 0.0011  mor+sham vs. mor+DBS: P = 0.0244 |
| 4H | control, n = 4 samples  OE, n = 4 samples | Unpaired t-test, two-tailed  t_6_ = 5.983, P = 0.0010 |
| 4I | OE+sham, n = 8 mice  control+DBS, n = 10 mice  OE+DBS , n = 9 mice | Two-way Repeated Measures ANOVA  Treatment: F_1, 24_ = 10.895, P = 0.0030  Test: F_2, 24_ = 2.947, P = 0.0720  Interaction: F_2, 24_ = 6.840, P = 0.0045  Bonferroni corrected post hoc comparisons  OE+sham vs. control+DBS  Test 1: 0.9999  Test 2: 0.0090  control+DBS vs. OE+DBS  Test 1: 0.9999  Test 2: 0.0021 |
| 4N | scramble, n = 4 samples  shRNA, n = 4 samples | Unpaired t-test, two-tailed  t_6_ = 2.478, P = 0.0479 |
| 4O | Scramble, n = 11 mice  shFGF 1, n = 14 mice | Two-way Repeated Measures ANOVA  Treatment: F_1, 23_ = 2.880, P = 0.1032  Test: F_2, 46_ = 6.719, P = 0.0028  Interaction: F_2, 46_ = 3.977, P = 0.0255  Bonferroni corrected post hoc comparisons  Scramble vs. shFGF 1  Test 1: >0.9999  Test 2: >0.9999  Priming: 0.0061 |
